# Therapeutic and protective effects of montelukast against doxorubicin-induced acute kidney damage in rats

**DOI:** 10.22038/ijbms.2019.33493.7996

**Published:** 2019-04

**Authors:** Evren Köse, Fatih Oğuz, Nigar Vardi, Mehmet Ediz Sarihan, Ali Beytur, Aytaç Yücel, Alaadin Polat, Nihat Eki̇nci̇

**Affiliations:** 1 Department of Anatomy, School of Medicine, Inonu University, Malatya, Turkey; 2 Department of Urology, School of Medicine, Inonu University, Malatya, Turkey; 3 Department of Histology-Embryology, School of Medicine, Inonu University, Malatya, Turkey; 4 Department of Emergency, School of Medicine, Inonu University, Malatya, Turkey; 5 Department of Anesthesiology and Reanimation, School of Medicine, Inonu University, Malatya, Turkey; 6 Department of Physiology, School of Medicine, Inonu University, Malatya, Turkey; 7 Department of Anatomy, School of Medicine, Erciyes University, Kayseri, Turkey

**Keywords:** Doxorubicin, Histology, Kidney, Leukotrienes, Oxidative stress, TBARS

## Abstract

**Objective(s):**

The current study was designed to investigate the therapeutic and protective effects of montelukast (ML) against doxorubicin (DOX)-induced acute kidney damage in rats.

**Materials and Methods:**

Thirty-five Wistar albino female rats were randomly divided into 5 groups as follows: Group I: Control; Group II: Control+ML; Group III: DOX; Group IV: DOX+ML; Group V: ML+DOX. At the end of the experiment, the kidney tissues of rats were collected. Thiobarbituric acid reactive substance (TBARS), reduced glutathione, superoxide dismutase (SOD), and catalase levels were determined from the kidney tissues. In addition, the kidney tissues were examined histologically.

**Results:**

DOX induced a significant increase in the kidney TBARS levels, whereas SOD contents significantly decreased when compared with the control group. On the other hand, ML administration before and after DOX injection caused signiﬁcant decreases in TBARS production and also increases in SOD levels. Histologically, the most remarkable damage was glomerulosclerosis and tubular changes in the DOX group. Moreover, marked tubular necrosis and swelling in tubular epithelial cells were observed in this group. Contrarily, although glomerulosclerosis was recognized as alleviated also in both DOX+ML and ML+DOX groups, the lesions did not completely ameliorate. However, treatment with ML after DOX injection was more effective than treatment with ML before DOX injection with respect to the protection of tubular structures.

**Conclusion:**

It was determined that ML treatment after DOX injection caused therapeutic effects against DOX-induced kidney damage. Thence, ML treatment is of some clinical properties for oxidative stress damage in kidney tissues.

## Introduction

Doxorubicin (DOX), an anthracycline antibiotic, has been used for the treatment of human neoplasms including leukemias, lymphomas, and solid tumors. The use of DOX is limited due to its side-effects; the most important are cardiotoxicity and nephrotoxicity (1-4). Though the toxicity of DOX on kidneys has not been clarified yet, it is believed that DOX-induced renal damage was caused by imbalanced oxidant-antioxidant systems (5, 6). Toxicity may be mediated through free radical formation, iron-dependent oxidative damage of biological macromolecules, membrane lipid peroxidation (LPO), and protein oxidation (7). The disturbance in oxidant-antioxidant systems, which has been demonstratedwith LPO and protein oxidation, results in tissue injury (8). 

Leukotrienes are a family of potent eicosanoid lipid mediators. They are synthesized from membrane phospholipids in response to cell activation. CysLTs (cysteinyl-leukotrienes) are produced from arachidonic acid through the 5-lipoxygenase (5-LO) pathway and act on the CysLT_1_ and CysLT_2 _receptors (9). A selective reversible CysLT1 receptor antagonist, montelukast (ML), is used in the treatment of asthma and reported to reduce eosinophilic inﬂammation in the airways (10-14). Sener *et al*. reported that bioactive metabolites of LTs had a pivotal role in the oxidative stress (15). Consistent with these important ﬁndings, we previously reported that ML had signiﬁcant antioxidant properties against methotrexate-induced renal and liver damages (16, 17).

To our knowledge, there has been no research regarding the protective and therapeutic effects of ML against DOX-induced acute kidney toxicity. Therefore, the current study was designed to investigate the therapeutic and protective effects of ML against DOX-induced acute kidney damage in rats.

## Materials and Methods

***Animals***

Forty Wistar albino female rats were housed in an air-conditioned room with 12 hr light and dark cycles, where the temperature (22±2 ^°^C) and relative humidity (65–70%) were kept constant. All experimental protocols were approved by the Experimental Animals Ethical Committee, School of Medicine, Inonu University, Malatya, Turkey.

***Experimental Protocol***

The rats were randomly divided into 5 groups as follows: Group I: Control; Group II: ML (Notta tb^®^ 10 mg, Sanovel, Turkey, 10 mg/kg daily for 10 days PO); Group III: DOX (Doxorubicin^®^ 50 mg, Farmar, Turkey, single dose 20 mg/kg IP); Group IV: DOX (single dose 20 mg/kg IP.)+ML (10 mg/kg daily for 10 days PO, 3 days after DOX injection); Group V: ML (10 mg/kg daily for 10 days PO)+DOX (single dose 20 mg/kg IP after the last dose of ML). 

On the 14th day, the rats in all groups were killed and the kidney tissues of rats were collected. The kidney tissues were placed into liquid nitrogen and stored at –70 ^°^C until assayed for thiobarbituric acid reactive substance (TBARS), reduced glutathione (GSH), superoxide dismutase (SOD), and catalase (CAT). The other kidney tissues were fixed at 10% formalin for histopathological analyses.

***Biochemical Analysis***

The levels of homogenized tissue TBARS, as an index of lipid peroxidation, were determined by thiobarbituric acid reaction using the method of Yagi (18). The product was evaluated spectrophotometrically at 532 nm, and the results were expressed as nmol/g tissue. 

The GSH content of the testis homogenate was measured at 412 nm using a known method (19). The GSH level was expressed as nmol/ml. 

Superoxide dismutase (SOD) activity was measured by the inhibition of nitroblue tetrazolium (NBT) reduction due to O_2_ generated by the xanthine/xanthine oxidase system (20). One unit of SOD activity was defined as the amount of protein causing 50% inhibition of the NBT reduction rate. The product was evaluated spectrophotometrically at 560 nm. The results were expressed as U/mg protein.

CAT activity of tissues was determined in accordance with the method explained by Aebi (21). The enzymatic decomposition of H_2_O_2_ was followed directly by the decrease in absorbance at 240 nm. The difference in absorbance per unit time was used as a measure of CAT activity. The enzyme activities were given in kU/mg protein.

***Histopathological evaluation***

The kidneys were fixed in 10% formalin, embedded in paraffin blocks, and sectioned. The sections of renal tissue were stained with hematoxylin-eosin (H-E) and periodic acid-Schiff (PAS) and examined by light microscopy in a blinded fashion. A semiquantitative morphometric score index was used to evaluate the degree of glomerulosclerosis. Sclerosis was defined as obliteration of glomerular capillary tuft and decrease of Bowman’s space.

Glomerulosclerosis scoring was performed by taking the severity and extent of the sclerotic lesion into consideration for each glomerulus. Thus, each glomerulus was assigned a score between 0 and 4 in the following way: 0 for normal glomeruli, 1 for sclerosis in ≤25% of the total area; 2 for sclerosis in 25%–50% of the total area; 3 for sclerosis in 50%–75% of the total area; and 4 for sclerosis in ≥80% of the total area. This analysis method of glomerulus was modified from Fujihara *et al. *(22). One hundred glomeruli were evaluated in each kidney, and the arithmetic mean of the sclerosis scores was accepted as the mean glomerulosclerosis score (MGS) for that rat. 

Tubular injury was defined as tubular necrosis, desquamation, tubular epithelial cell swelling, and loss of the brush border. The following semiquantitative score was used: score 0=no tubular injury; score 1= 0–25% of the tubules were injured; score 2=25–50% of the tubules were injured; score 3=50–75% of the tubules injured; and score 4 = >75% of the tubules were injured. For each specimen, 10 microscopic fields were analyzed under a 20X objective per animal. The sections were examined using a Leica DFC 280 light microscope. 

***Biostatistical Analysis***

The data were expressed as either median (min-max) value. Normality distribution was assessed using the Shapiro-Wilk test. The non-normally distributed data were compared by the Kruskal Wallis H test between the groups. When significant differences were determined, multiple comparisons were carried out using the Mann Whitney U test with Bonferroni correction. *P*<0.05 values were considered as significant. IBM SPSS statistics version 25.0 for Windows was used for statistical analyses.

## Results

***Biochemical results***

The kidney TBARS, GSH, SOD, and CAT levels for ML and DOX-treated rats were presented in Table 1. It was determined that DOX induced a significant increase in the kidney TBARS levels, whereas SOD contents significantly decreased when compared with the control group (*P*<0.05). On the other hand, ML administration before and after DOX injection caused signiﬁcant decreases in TBARS production and also increases in SOD levels (*P*<0.05). There were no statistically significant differences for GSH and CAT levels. On the other hand, the levels of GSH and CAT of the DOX-ML group were higher than those of the ML-DOX group.

**Figure 1:**
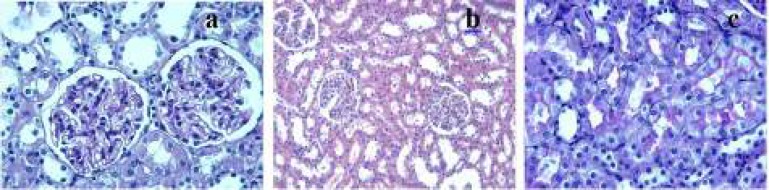
Control group

(a) Renal glomeruli and (b) tubules are intact. (c) Brush border is visible. (a) and (c) PAS X40, (b)H-E X20

PAS: periodic acid-Schiff, H-E: hematoxylin-eosin

**Figure 2:**
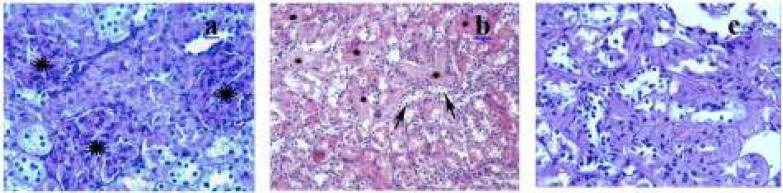
DOX group

(a) Obliteration of glomerular capillary tuft and presence of glomerular tuft capsular adhesion compared to the control group can be observed in the glomerulus (b) tubules show marked necrosis (*) and swelling in tubular epithelial cells (arrows) (c) notice lost brush border. (a) and (c) PAS X40, (b) H-E X20

DOX: Doxorubicin, PAS: periodic acid-Schiff, H-E: hematoxylin-eosin

**Figure 3:**
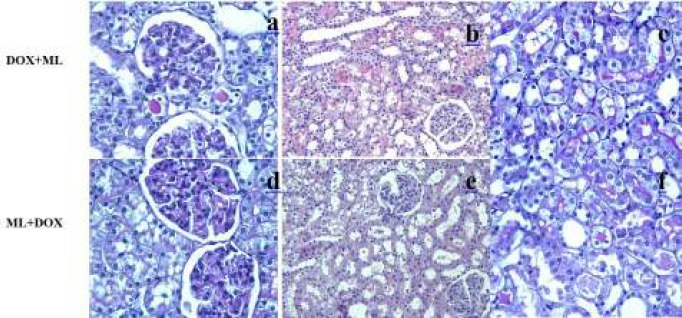
DOX+ML and ML+DOX groups

(a) and (d) there is a prominent reduction in the degree of glomerulosclerosis according to the DOX group. Bowman’s space is visible (b) and (e) moderate tubular damage such as desquamation of tubular epithelial cells are observed. (c) the view of brush border is almost similar to the control group (f) the loss of brush border is marked in affected tubules. (a,d) and (c,f) PASX40, (b,e) H-E X20

DOX: Doxorubicin, ML: montelukast, PAS: periodic acid-Schiff, H-E: hematoxylin-eosin

**Table 1:** The levels of biochemical parameters of all groups

**Parameters**	**Control**	**ML**	**DOX**	**ML-DOX**	**DOX-ML**
MDA	197,1 (107,1-222,7)	236,5 (104,9-250,1)	255,6 (194,3-308,2)^a^	159,8 (105,7-267,1)	143,2 (119,6-185,9)
SOD	0,396(0,35-0,47)	0,395 (0,35-0,51)	0,269 (0,21-0,4)^ b^	0,415 (0,29-0,55)	0,51 (0,31-0,64)
CAT	377,25±34,29	364,91±34,29	301,17±102,16	330,21±93,87	403,57±87,20
GSH	2,26±0,27	2,58±0,36	2,33±0,46	1,67±0,26	2,49±02,99

a Significantly increased compared with control, ML-DOX, and DOX-ML

b Significantly decreased compared with other groups

**Table 2:** The comparison of severity of glomerulosclerosis among groups

	**Mean glomerulosclerosis score**				
**Groups**	0	1	2	3	4
Control	95.9±0.5	4.1±0.5	0.0±0.0	0.0±0.0	0.0±0.0
ML	99.3±0.2	0.6±0.2	0.0±0.0	0.0±0.0	0.0±0.0
DOX	0.0±0.0^a^	2.1±0.3	7.3±0.3^b^	45.1±0.8^b^	45.37±0.6^b^
DOX-ML	10.0±0.1^a^,^c^	40.1±0.4^b^,^c^	33.6±0.6^b^,^c^	13.2±0.7^b^,^d^	3.0±0.2^d^
ML-DOX	18.0±0.2^a^,^c^	32.6±0.7^b^,^c^,^f^	32.±0.6^b^,^c^,^e^	17.37±0.7^b^,^d^	0.0±0.0 d,f

a Significantly decreased when compared with the control group, *P* =0.00.

b Significantly increased when compared with the control group, *P* =0.00.

c Significantly increased when compared with the DOX group, *P* =0.00.

d Significantly decreased when compared with the DOX group, *P* =0.00.

e Not significant when compared with the DOX-ML group, *P* =0.6.

f Significantly decreased when compared with the DOX-ML group, *P* =0.00.

**Table 3:** The comparison of severity of renal tubular injury between groups

**Groups**	**Tubular Injury**
Control	0.4±0.2
ML	0.5±0.2
DOX	3.2±0.3^a^
DOX-ML ML-DOX	1.6±0.6^a^,^b^ 2.4± 0.2^a^

a Significantly increased when compared with the control group, *P* =0.01.

b Significantly decreased when compared with the DOX group, *P* =0.005.

***Histological Results***

The control group showed normal or slight changes such as epithelial desquamation. Glomeruli and brush border were observed as intact (Figures 1a, 1b, and 1c). The ML group exhibited similar morphological changes as observed in the control group. The most remarkable damage was glomerulosclerosis and tubular changes in the DOX group. Glomeruli revealed obliteration of glomerular capillary tuft and presence of glomerular tuft capsular adhesion (Figure 2a). Moreover, marked tubular necrosis and swelling in tubular epithelial cells were observed in this group (Figure 2b). The widespread loss of the brush border was observed in the affected tubules (Figure 2c). On the other hand, although glomerulosclerosis was recognized as alleviated also in both DOX+ML and ML+DOX groups, the lesions did not completely ameliorate. (Figures 3a and 3d). The appearance of glomeruli was similar in these groups. However, treatment with ML after DOX injection was more effective than treatment with ML before DOX injection with respect to the protection of tubular structures (Figures 3b and 3e). But, no difference was found significant statistically between the two groups in terms of tubular injury (*P*>0.05). Brush border was relatively intact in the DOX+ML group according to the ML+DOX group (Figures 3c and 3f). Using semiquantitative scoring methods, the mean values of the glomerulosclerosis and tubular damage were determined and given in Tables 2 and 3.

## Discussion

In the current research, the potentially harmful effects of DOX on the kidneys were examined biochemically and histologically. DOX is used as a chemotherapeutic agent in various leukemias and lymphomas (1). Despite its beneficial effects, DOX has considerable cardiotoxic and nephrotoxic harmful effects (2, 3, 23). DOX-induced cardiotoxicity and nephrotoxicity have been investigated in many biomedical studies. It is reported that the use of DOX results in increased production of free radicals that react rapidly with lipids causing Lipid peroxidation (LPO) (24). Increased LPO is measured in terms of TBARS levels. In the present research, DOX-treated rats showed an increased level of TBARS and a decreased level of SOD compared to control rats. TBARS, a stable metabolite of the free radical-mediated lipid peroxidation cascade, is used widely as a marker of oxidative stress and lipid layer destruction (25). Elevated TBARS levels show that DOX has caused oxidative stress and the formation of free radicals in the kidney tissue. SOD has a significant role against oxidative damage caused by free radicals (26) and converts superoxide ion (O_2_^-^) to hydrogen peroxide (H_2_O_2_). A clinical study examined the effects of nicotinamide against nephrotoxicity induced by DOX and reported that antioxidant and anti-inflammatory properties of NAD could decrease the nephrotoxicity induced by DOX (27). In accordance with the mentioned study (27), experimental findings of this study have indicated that the therapeutic and protective effects of ML administration can impair DOX-induced acute renal damages in rats. This important inference from the experiments has been confirmed based on histological and biochemical findings of the treatment of ML.

Another objective of the current research was to investigate the possible therapeutic and protective effects of ML against DOX-induced acute kidney damage in rats. ML is employed to treat asthma and to alleviate the symptoms of seasonal allergies and can decrease eosinophilic inflammation in the respiratory tract (28). In this study, ML treatment before and after DOX injection prevented possible oxidative stress damage in kidney tissues. These important findings were achieved from the biochemical data owing to the signiﬁcant decreases of TBARS production and the signiﬁcant increases of SOD levels. According to the histological results, renal damage decreased in a similar way to the biochemical results. The treatment with ML after DOX injection in the reduction of tubular injury was more successful as compared with the treatment with ML before DOX injection. In a clinical trial similar to our hypothesis, the potential protective effect of ML on renal ischemia/reperfusion (I/R) damage was evaluated and was concluded that ML maintained renal tissue by improving oxidant–antioxidant balance, which was proved with biochemical and histopathological findings (10). Another experimental research examined the probable protective effect of ML towards oxidative damage in kidney tissues and indicated that ML is capable of protecting kidney tissues from oxidative damage (29). In our previous study, we designed a different experimental model investigating the protective and therapeutic effects of ML against amikacin-induced acute kidney injury. We showed that ML administration after amikacin injection decreased the oxidative injury (28).

With respect to DOX administration, a recent study has dealt with the current schemes for primary and secondary prevention targeting by contrasting the commencement of early and late DOX-induced cardiotoxic events (2). Additionally, the protective effects of dioscin were administrated on the DOX-induced nephrotoxicity by correcting FXR-mediated inflammation and oxidative stress (4). In this respect, DOX-induced applications in biomedical studies keep up to date from the clinical point of view.

## Conclusion

It was determined that ML treatment after DOX injection caused therapeutic effects against DOX-induced kidney damage. Thence, ML treatment has some clinical applications for oxidative stress damage in kidney tissues.
